# Assessing the Impact of COVID-19 Prevention Measures on Adolescent Growth in Italy

**DOI:** 10.3390/healthcare11142101

**Published:** 2023-07-24

**Authors:** Luciana Zaccagni, Natascia Rinaldo, Gianni Mazzoni, Simona Mandini, Sabrina Masotti, Stefania Toselli, Federica De Luca, Emanuela Gualdi-Russo

**Affiliations:** 1 Department of Neuroscience and Rehabilitation, Faculty of Medicine, Pharmacy and Prevention, University of Ferrara, Corso Ercole I d’Este 32, 44121 Ferrara, Italy; luciana.zaccagni@unife.it (L.Z.); gianni.mazzoni@unife.it (G.M.); simona.mandini@unife.it (S.M.); sabrina.masotti@unife.it (S.M.); federica.deluca@unife.it (F.D.L.); 2 Center for Exercise Science and Sports, University of Ferrara, 44123 Ferrara, Italy; 3 Department for Life Quality Studies, University of Bologna, 47921 Rimini, Italy; stefania.toselli@unibo.it

**Keywords:** adolescence, COVID-19, lockdown, obesity, body image perception, somatic growth, sedentary lifestyle

## Abstract

COVID-19 infection has caused increased morbidity and mortality worldwide. Several strategies have been adopted around the world to prevent its spread. Italy underwent a long lockdown for face-to-face educational activities, which were replaced with online classes. This longitudinal study aimed to analyze the effects of COVID-19 prevention measures on physical growth and body image perception in a sample of Italian adolescents who experienced the pandemic-induced lockdown in 2020. In particular, we wished to ascertain how lifestyle changes had affected their growth rates and health. Special attention was paid to increases in adiposity indicators (BMI, waist circumference, waist-to-height ratio) and weight caused by reduced physical activity, and consequent possible dissatisfaction with body image. We assessed the impact of school closures by comparing the annual growth rate and body image perception changes of adolescents (n = 60; age = 11.3 ± 0.4 years) who experienced this isolation with those in the following years who did not experience these restrictions (n = 68; age = 11.4 ± 0.3 years). As a consequence of the lockdown, our results indicate a greater annual growth rate in weight and other indices of adiposity (*p* < 0.05). As the virus is continuing to evolve and propagate, larger population studies can verify and confirm our findings. In promoting health policy to prevent the ongoing prevalence of obesity in adolescents, an accurate assessment of whether the increase in obesity rates during the pandemic is to be considered a temporary trend is highly recommended.

## 1. Introduction

COVID-19 is an infectious disease with a high level of infectiousness and mortality caused by the Severe acute respiratory syndrome coronavirus 2 (SARS-CoV-2 virus) that emerged in late 2019. The World Health Organization (WHO) stated the COVID-19 outbreak was a global pandemic on 11 March 2020 [1]. Just one year after the first COVID-19 case was identified, in December 2020, the first COVID-19 vaccinations were administered [2]. December 27 was the symbolic start (Vaccine Day) of the COVID-19 vaccination campaign throughout Europe, including Italy. Unlike adults, vaccination for children aged 12 years and older did not start in Italy until 2021 (in May with the Comirnaty vaccine and in July with Spikevax). Preventive measures taken around the world to keep this pandemic under public health control have included general strategies. Among these, social distancing, stay-at-home orders, curfews, travel restrictions, and isolation are particularly effective in preventing COVID-19 when mandatory and undertaken early [3].

The positive effect of social distancing, such as school closures, has been reported in the literature [4]. Many countries worldwide experienced the suspension of face-to-face teaching during the COVID-19 pandemic, such that 95% of the world’s student population seems to be involved [5]. Italy had one of the highest prevalences of this disease, probably due to the advanced average age of the population [6]. During this study, as of March 2020, more than 27,000 people were found to have COVID-19, with 2158 deaths [6]. Currently (22–28 May 2023), the incidence rate of COVID-19 in the Emilia-Romagna region is 17.4 cases per 100,000 people [7]. In the year 2020, as a result of the pandemic, Italian schools and any sports or aggregation activities were forced to close from March 5 to the end of the school year—this closure had been brought forward to February 24 in the Emilia-Romagna region for precautionary purposes. Due to the confinement measures, teaching activities continued, when possible, at a distance through digital technologies (online classes). This condition heavily impacted interpersonal relationships, resulting in physical emptiness and emotional distress [8]. We assume that this emergence may also have affected the children’s and adolescents’ physical health due to lack of physical activity, as well as possible emotional eating with the appearance or worsening of nutritional disorders. Confirming this, an Italian survey carried out during lockdown with interviews with adults found that about half of the interviewees were anxious about their eating habits, consuming comfort food and increasing food consumption to improve their feelings [9].

Lifestyle and eating behavior modifications due to social isolation during the pandemic have been pointed out as risk factors for the rise in obesity prevalence [10]. Sedentary behavior is associated with increased overweight/obesity [11]. According to a review of the literature conducted in 2016 [12], 80% of obese adolescents are likely to become obese adults. A questionnaire survey found that the isolation of the pandemic caused Dutch children to be less physically active and to spend more screen time during and after school closures [13]. Given the increased risk of morbidity and mortality, adolescent obesity is a serious public health problem and an increasing social problem worldwide [12,14,15].

Although it appears that the restrictive measures imposed by the lockdown harmed psychological well-being [9], there is very little information on the effects of the lockdown caused by the pandemic on the growth of healthy children and adolescents. This was certainly not caused by a lack of interest, but rather by the difficulty of access in public schools during the pandemic. Although there is no lack of studies showing the risk of weight gain in children with overweight or obesity followed in medical centers during the pandemic [16], it is essential to gather all the information possible to ascertain how changes in healthy adolescents’ daily lives with isolation measures and social estrangement may have impacted their growth process and body image (BI) perception. An important finding from the literature is an increased rate of obesity during the pandemic in Korean children and adolescents [17,18], particularly in males [19], and Turkish children under age 11 [10]. The different gain in weight between sexes with a significant increase in the prevalence of overweight and obesity in males was interpreted with the increased sitting time in the male sex, in contrast to females, for whom a decrease was observed [19]. This trend has also been confirmed in Western Europe [16,20]. Previous studies were generally aimed at assessing the incidence of overweight and obesity during the pandemic through cross-sectional approach research.

A large ongoing research project aimed at evaluating the health and nutritional status, behaviors, and lifestyle of adolescent students in the Emilia-Romagna region (Northern Italy) [21,22] provided us with the opportunity to carry out the present study by analyzing the growth of a sample of adolescents evaluated before and after the Italian lockdown in comparison with the growth of peers evaluated subsequently. This study was specifically aimed at assessing the growth pattern of anthropometric traits, particularly adiposity indices, in response to lifestyle changes caused by the lockdown imposed to limit the spread of the pandemic in Italian adolescents enrolled in secondary school. The assessment of anthropometric traits represents a noninvasive and indirect quantitative method of measuring body composition. In particular, adiposity indices allow the prediction of excess body fat that can have serious health consequences: excess fat in the abdominal region (central obesity) is a better predictor of health risk than BMI [23]. Taking into account the lifestyle changes, this study is one of the first efforts to measure the effects of the COVID-19 lockdown on adolescent growth by a longitudinal research design in Italy.

## 2. Materials and Methods

### 2.1. Study Design and Participants

This is an observational prospective study. It analyzed some of the data we collected with a longitudinal design in a public middle school in the city of Ferrara (Emilia-Romagna region, Italy), chosen by a convenience criterion. Participants, sixth-grade schoolchildren, were recruited through official letters sent by the school. All schoolchildren who had written informed consent signed by their parents or guardians and agreed to participate were enrolled in the study. Subjects could withdraw their consent to participate at any time. In particular, we excluded 20 children as they did not have signed consent. The baseline survey was carried out in November 2019, before the announcement of school closures in Italy in 2020. After the study was temporarily suspended due to COVID-19, a repetition of the measurements on the same children was possible only one year later, allowing us to perform pre- and post-lockdown examinations. In total, this study analyzed the growth of 128 schoolchildren divided as specified below. Of the 61 children examined before the beginning of the pandemic, 60 were re-examined, making up the study sample (lockdown group). As a control group, 68 children who started sixth grade in 2020 were selected and re-examined in 2021. In these surveys, no missing values were found in anthropometric measurements or BI questions. The study was conducted in accordance with the Declaration of Helsinki (64th WMA General Assembly, Fortaleza, Brazil, October 2013), and all procedures were approved by a local University Bioethics Committee (Bologna; Ethical Approval No. 2.18). Before collecting anthropometric measurements, participants answered some basic socio-demographic questions (Supplementary Material Table S1). Any identifying information about the participant was removed during the processing of the data (reported as means or frequencies) so as to ensure the complete anonymity of each subject.

### 2.2. Anthropometric Measurements

All anthropometric measurements were taken by the same experienced operator (L.Z.) according to traditional anthropometric methods [24,25,26,27]. Repeated measurements included weight, stature, and waist circumference (WC). Participants were measured in the morning while wearing light indoor clothes and barefoot. Body weight was measured to the nearest 0.5 kg using a mechanical scale (SECA, Basel, Switzerland). Stature was measured to the nearest 0.1 cm with an anthropometer (Magnimeter, Raven Equipment Ltd., Dunmow, UK) on participants with the head oriented according to the Frankfurt plane. WC was measured to the nearest 0.1 cm with a non-stretchable metric tape (DKSH, Zurich, Switzerland) halfway between the last rib and the iliac crest.

We calculated the Body Mass Index (BMI) as weight/stature^2^ (kg/m^2^) for the assessment of the participants’ weight status according to Cole et al. cutoffs [28,29]. As a result, the participants were classified as underweight, normal weight, overweight, and obese. Due to the small number of obese subjects, the overweight and obese categories were merged. Dividing the WC with the stature, we obtained the relative index (WHtR) that is assumed to indicate increased health risk for values ≥0.5 [30]. This cutoff point can be applied regardless of age and sex in children [31]. Finally, we assessed participants’ risk of central obesity based on their WC using the cutoffs recently proposed by Xi et al. [32]. We calculated the annual growth rate by the ratio of the difference between two repeated measures and the interval of time (1 year).

### 2.3. Body Image Perception

The feel and ideal BI of participants were assessed using the Childress et al. scale [33], consisting of eight silhouette drawings of female or male figures that are child adaptations of adult drawings developed by Stunkard et al. [34]. The eight silhouettes, having equal stature, were ordered incrementally from an emaciated figure to an obese one. The participants were asked to indicate which figure best represented their actual body size and which would be their ideal. Specifically, each adolescent was required to choose male or female silhouettes in accordance with their gender (see instructions in Supplementary Material Table S2). This assessment was carried out on the same subjects in both surveys.

The FID score made it possible to assess the BI dissatisfaction as Feel minus Ideal Discrepancy. The higher the score at both signs, the greater the dissatisfaction. In the case of a positive score, the perceived dimensions are greater than the ideal, while a negative score indicates that the perceived dimensions are smaller than the ideal. Only in the case that the score is equal to 0 (feel figure = ideal figure) is no dissatisfaction present.

### 2.4. Statistical Analyses

The normality of the variables was checked using the Kolmogorov–Smirnov test: anthropometric traits were normally distributed, unlike the body image perception variables. For descriptive analysis, continuous variables were described by mean and standard deviation (SD), and categorical variables were described by frequency and percentage. Comparisons of anthropometric traits and growth rate between groups (lockdown vs. control) and survey (1st vs. 2nd) were performed using an independent *t*-test; comparisons of BI traits through the non-parametric Mann-Whitney U test. The chi-squared test was applied to assess the difference in frequency between the two groups (lockdown and control) and the two surveys.

All statistical analyses were carried out using STATISTICA software, version 11 (StatSoft, Tulsa, OK, USA), and the significance level for all statistical tests was set at *p* < 0.05.

## 3. Results

Table 1 shows the baseline anthropometric characteristics of the sample stratified by sex and groups (lockdown and control).

It is important to note that at baseline, no significant differences resulted from the comparison between participants who experienced the Italian lockdown (lockdown group) and controls (Table 1). At baseline, three girls of the female lockdown group (12% of the subsample), and seven girls of the control group (21.9% of the subsample) had WC higher (χ^2^ = 0.387, *p* = 0.534) than cutoffs for adult central obesity [4]; two girls of the former group (8% of the subsample), and five girls of latter group (15.6% of the subsample) had a WHtR value higher (χ^2^ = 0.215, *p* = 0.643) than the critical value (0.5). In the male sex, two boys of the lockdown group (5.7%), and three boys (8.3%) of the control group exceeded the WC cutoffs (χ^2^ = 0.001, *p* = 0.974); three boys of the lockdown group (8.6%), and nine control boys (25%) exceeded the WHtR cutoff (χ^2^ = 2.514, *p* = 0.113). There was no difference in at-risk subjects between the sexes and groups for WC (χ^2^ = 0.038, *p* = 0.846) and WHtR (χ^2^ = 0.137, *p* = 0.712).

Similar results are reported for BI perception and dissatisfaction. No differences resulted between adolescents that experienced lockdown between the two surveys and controls. The level of dissatisfaction did not differ between adolescents of the two groups, with lower mean values in males in comparison to females. All the groups had positive values of FID, meaning a desire to become thinner.

As regards weight status (WS) (Table 2), no significant differences were found between the two groups at baseline in both sexes (females: χ^2^ = 0.147, *p* = 0.929; males: χ^2^ = 3.609, *p* = 0.165) and within the same group of the same sex between the two surveys (female lockdown group: χ^2^ = 0.097, *p* = 0.953, female controls: χ^2^ = 0.259, *p* = 0.879; male lockdown group: χ^2^ = 1.621, *p* = 0.445; male controls: χ^2^ = 0.941, *p* = 0.625). Concerning changes in the WS category between the two surveys, three girls of the “lockdown” group changed their WS during the lockdown (12%): one from normal weight to underweight and two from underweight to normal weight. In the female control group, three decreased their WS, and three increased it (18.8%). As regards the male group, six boys out of 35 changed WS during lockdown (17.1%), two decreasing and four increasing their WS; on the other hand, among boys in the control group, six decreased their WS, and two increased it (22.2%).

No significant differences in the annual growth rate of the anthropometric variables emerged in the subsample of females (Table 3), although higher mean values were found in the lockdown group than in the control group. Boys of the two groups did not have significantly different longitudinal growth; weight, BMI, WC, and WHtR increased significantly rapidly in the group subjected to lockdown between the two surveys. Moreover, there was a decrease in BMI and WHtR rate in males of the control group. Specifically regarding WC, boys at risk of central obesity doubled in the lockdown group, from 2 in the first survey to 4 in the second one, while in the control group, they decreased by a third, from 3 to 2; concerning WHtR, the boys with increased health risk (WHtR ≥ 0.5) more than doubled in the lockdown group, from 3 in the first survey to 7 (20%) in the second survey, while they became one third, from 9 in the first survey to 3 (8%) in the second one, in the control group.

As regards the changes in BI perception, a decrease in dissatisfaction was highlighted in girls of both groups, whereas boys resulted in higher values of dissatisfaction in the second survey.

## 4. Discussion

The present study found that the yearly growth velocities in weight, WC, BMI, and WHtR of Italian sixth-grade adolescents increased during the COVID-19 lockdown compared with same-sex control peers measured in the school year after the lockdown. However, these trends were found to be statistically significant only in the male sex. The general pattern observed for the lockdown group in comparison to controls is consistent with our first hypothesis that the greatest increases in adiposity indices may be found in the group exposed to an adverse (obesogenic) environment. How, then, can the differences between the sexes be explained? This trend is not surprising since the higher eco-sensitivity of males compared with females is well known. Indeed, numerous previous studies show the greater stability of the female sex in response to different environmental changes both in developmental age [35,36,37] and adulthood [38,39]. Changes in living conditions can induce different reactions in males and females leading to different levels of adaptation and health status [40,41] consistent with the “fragile male” theory [42], which assumes greater sensitivity of males to suboptimal environments throughout growth and development. The fact that females are better “canalized” in growth than males would be attributable, according to Tanner [43], to their possession of two X chromosomes or their greater power of homeorhesis.

As a confirmation of the different eco-sensitivities of the two sexes resulting from lifestyle changes before and during the COVID-19 outbreak, a higher incidence of obesity in the male sex was found in Korean adolescents [19]. In the present study, with a longitudinal design, the percentage of overweight/obese subjects increased by 11.4% in the male sex compared to 4% in the female sex (almost three times more). Other previous studies, generally with a cross-sectional design, have also shown an increase in obesity during the pandemic [44,45]. In particular, consistent with our findings, the prevalence of obesity increased from 13.7 to 15.4% in US children [44] and from 11.5 to 12.7% in Korean adolescents [19] during the pandemic. General lifestyle, psychosocial, and environmental changes resulting from the pandemic have given rise to fast and widespread weight gain in populations all over the world, so much so that this phenomenon has been named “covibesity” [46], and young people who have experienced it “Generation Corona” [47]. In addition to an alteration in eating habits with stress and emotional disturbance, homeschooling and TV use increased screen time [46].

In Italy, the government has implemented strict measures to contain the COVID-19 pandemic with a decree (2020) [48] that modified the living habits of Italians by imposing a long lockdown. Our findings can be a direct effect of these lifestyle changes. First, a forced sedentariness of the entire population including adolescents was determined: any physical activity they had previously carried out during the home-to-school route or school and extra-school hours were suppressed; organized sports activities were also suspended in Italy. Confirming this, a cross-sectional study conducted in Italy on undergraduate students by electronic questionnaires showed that sedentary behaviors increased and physical activities decreased during the lockdown [49]. Second, the forced stay-at-home increased the consumption of comfort foods [9]. An increase in unhealthy eating habits, such as snack consumption, was also reported during the pandemic [9,50]. A previous study on Italian adolescents found an increase in processed meat, bread, pizza, and bakery products in particular, aided by increased boredom in staying home during the lockdown [49]. Another factor that may have promoted a greater food intake was the increased screen time: it was found that children and adolescents increased their exposure to electronic devices as much as four hours/day during this period [51]. A study on adolescent’s dietary habits conducted through an anonymous online questionnaire during the pandemic in Spain, Italy, Brazil, Colombia, and Chile showed that girls consumed significantly more fruits and vegetables, and less sugar-sweetened beverages than boys during confinement due to COVID-19 [52]. It is plausible that the different eating habits of the two sexes may also have influenced the anthropometric differences we found in our study as sugary drinks and sweet foods can have negative health effects including overweight and obesity. In general, the results obtained confirm the influence exerted on body characteristics by the change of lifestyle behaviors with increases in energy intake over energy expenditures as suggested in a study conducted in Greece [53]. The last study, carried out through questionnaires and considering self-reported weight, reported that body weight increased by 35% in children and adolescents as a consequence of the lockdown. However, we should mention that during the pandemic, in addition to negative changes in eating (e.g., snacking, eating when watching television), some positive changes (e.g., participation in home cooking, eating with others) were also detected [54].

There are a number of factors that can influence the body composition and weight status of children and adolescents, such as family habits and socio-economic variables. Nuvoli, analyzing the eating habits of Italian children and adults, reported that children who eat several meals a day with their family are more likely to be of normal weight [55]. Among lifestyle habits, sleep duration and quality also play an important role in weight status [56,57]. Lower sleep quality during the period of confinement was also confirmed by a study of Italian university students [58].

In addition to excessive inappropriate food consumption, sedentariness and physical inactivity can lead to overweight/obesity. The importance of physical activity to the overall health of the individual is well known. Adolescence also represents a delicate phase for mental health [59], and possible negative health consequences of inactivity may include those related to BI: unlike positive BI, negative BI is generally associated with lack/reduction of physical activity and sports practice [60]. Participation in physical activities and sports exerts a protective effect against BI-related issues by increasing body satisfaction. In a questionnaire survey conducted in Europe, Kovacs et al. [61] showed a general insufficient level of physical activity and unhealthy screen time in children in reaction to the COVID-19 pandemic. A reduction or, as in the case of the Italian lockdown, the total suspension of such activities may exert mental health consequences during adolescence. However, considering changes in BI perception and dissatisfaction before and after the lockdown, no statistically significant difference resulted in comparison with controls. While generally confirming the girls’ lower body appreciation [21,59,62], slight changes in BI ideals and BI dissatisfaction were found in both sexes at the second survey consistently with increased indicators of adiposity during the lockdown. Thus, these adolescents were aware of the increased weight, but this did not significantly affect their body dissatisfaction. Being accepted by peers is associated with BI in adolescence [63,64], but in this case, we assume that the isolation from other peers during lockdown protected these adolescents from BI concerns. Despite some fluctuations in perception from the BI reported in the literature [65], we confirm that the BI perception of the adolescents examined maintained its traditional stability during the lockdown in Italian adolescents.

This study has some strengths and weaknesses that we would like to comment on. As regards the strengths, this was the first study to examine the potential effects of the COVID-19 lockdown on indicators of adiposity and BI dissatisfaction in Italian adolescents. In addition, our survey included the use of anthropometric indicators of adiposity measured directly before and after the COVID-19 lockdown on the same sample (longitudinal study), unlike other studies that, due to the limitations imposed by the pandemic, generally made use of questionnaires to collect anthropometric data (see, for example, [53]). The main weakness of the study is the small sample size and the non-probability sampling method used. Moreover, we do not have precise information on screen time or exercise practice at home by the children examined. However, the general living conditions were very similar for the entire Italian population due to the strict rules imposed by the government during the COVID-19 lockdown. Another limitation is that we did not collect data on the socio-economic status and family habits of each participant. Finally, a further limitation of the research is that the controls were not analyzed before the epidemic, but one year after the lockdown ended. Although, therefore, these adolescents were not affected as closely as the sample on whom we tested the effects of the lockdown, they too experienced this phenomenon previously during elementary school.

## 5. Conclusions

The lockdown was a major measure to prevent the spread of the pandemic when it was still relatively unknown. This study found that these preventive actions against the spread of COVID-19 in Italy had a negative impact on adolescents’ health, especially among males, with an increase in general and central adiposity, while no impact was detected on BI perception and dissatisfaction. Increases in overweight/obesity and central obesity found in adolescents submitted to lockdown have serious implications because of the risk of long-term metabolic diseases. The strength of this impact depended interestingly on gender: girls appeared to be less sensitive than boys to lifestyle changes. Current knowledge regarding surveillance measures on the spread of COVID-19, as well as the end of the pandemic (declared on 5 May 2023), will likely enable the avoidance of further lockdowns, a source of stress and cause of unhealthy behavior by adolescents. Innovative targeted and effective interventions against this or other new pandemics will avoid deleterious effects on adolescents’ health. In particular, special strategies to avoid infections, from masks and sanitation to individual and group testing and vaccination, should be developed and implemented as much as possible to avoid total school closures and the resulting unhealthy lifestyles, as in the sample examined in this study.

## Figures and Tables

**Table 1:** Comparison of baseline anthropometric characteristics between lockdown and control groups by sex.

	Lockdown N = 25		Control N = 32		
	Mean	SD	Mean	SD	*p*
*FEMALES*					
**Anthropometric traits**					
Stature (cm)	150.98	7.22	152.41	5.23	0.390 ^a^
Weight (kg)	44.76	7.80	46.66	11.64	0.487 ^a^
BMI (kg/m^2^)	19.63	3.12	20.03	4.55	0.711 ^a^
WC (cm)	64.85	6.53	65.98	9.31	0.608 ^a^
WHtR	0.43	0.05	0.43	0.06	0.870 ^a^
**Body image perception**					
Feel figure	4.16	1.11	4.07	1.31	0.627 ^b^
Ideal figure	3.20	0.87	3.52	0.74	0.274 ^b^
FID	0.96	1.14	0.55	1.21	0.094 ^b^
*MALES*	N = 35		N = 36		
**Anthropometric traits**					
Stature (cm)	147.19	6.46	149.01	8.96	0.331 ^a^
Weight (kg)	41.00	7.97	44.90	10.48	0.082 ^a^
BMI (kg/m^2^)	18.81	2.85	20.13	3.98	0.114 ^a^
WC (cm)	65.93	7.78	69.60	9.84	0.086 ^a^
WHtR	0.45	0.05	0.47	0.07	0.143 ^a^
**Body image perception**					
Feel figure	4.09	1.20	4.55	1.30	0.152 ^b^
Ideal figure	3.71	0.57	4.03	1.30	0.227 ^b^
FID	0.37	1.17	0.42	1.32	0.936 ^b^

BMI: body mass index; WC: waist circumference; WHtR: waist-to-height ratio; FID: Feel minus Ideal Discrepancy. ^a^ comparison performed through *t*-test for independent samples. ^b^ comparison performed through U-Mann-Whitney non-parametric test.

**Table 2:** Composition of two groups by weight status categories, sex, and surveys.

	Lockdown N = 25		Control N = 32	
	I Survey	II Survey	I Survey	II Survey
*FEMALES*				
Underweight	1 (4.0)	1 (4.0)	2 (6.3)	3 (9.4)
Normal weight	17 (68.0)	16 (64.0)	21 (65.6)	21 (65.6)
Overweight/obese	7 (28.0)	8 (32.0)	9 (28.1)	8 (25.0)
*MALES*	N = 35		N = 36	
Underweight	2 (5.7)	3 (8.6)	3 (8.3)	2 (5.6)
Normal weight	26 (74.3)	21 (60.0)	19 (52.8)	23 (63.9)
Overweight/obese	7 (20.0)	11 (31.4)	14 (38.9)	11 (30.6)

**Table 3:** Comparison of anthropometric data annual growth rate and change in FID between lockdown and control groups by sex.

	Lockdown		Control		
	Mean	SD	Mean	SD	*p*
*FEMALES*					
Stature rate (cm/year)	5.68	2.26	4.69	2.36	0.117
Weight rate (kg/year)	5.37	3.27	4.00	4.44	0.203
WC rate (cm/year)	2.77	3.25	1.48	5.48	0.302
BMI rate ((kg/m^2^)/year)	0.84	1.31	0.48	1.77	0.400
WHtR rate (cm/cm/year)	0.002	0.021	−0.004	0.035	0.469
Change in FID	−0.20	0.58	−0.10	0.49	0.509
*MALES*					
Stature rate (cm/year)	7.00	2.40	7.07	2.99	0.910
Weight rate (kg/year)	6.80	3.30	4.31	3.45	**0.003**
WC rate (cm/year)	4.18	3.60	0.84	3.03	**0.000**
BMI rate (kg/m^2^/year)	1.13	1.15	−0.09	1.67	**0.001**
WHtR rate (cm/cm/year)	0.007	0.026	−0.016	0.025	**0.000**
Change in FID	0.09	0.78	0.24	1.48	0.592

BMI: body mass index; WC: waist circumference; WHtR: waist-to-height ratio; FID: Feel minus Ideal Discrepancy. Significant *p*-values are presented in bold.

## Data Availability

Data are available upon request due to ethical restrictions regarding participant privacy. Data requests may be sent to the corresponding authors.

## Supplementary Materials

The following supporting information can be downloaded at: https://www.mdpi.com/article/10.3390/healthcare11142101/s1, Table S1: Basic socio-demographic information collected during the survey; Table S2: Instructions to assess the body image perception using the scale of silhouettes developed by Childress et al. [33].
