# Supplementary material for: Assessing the Impact of COVID-19 Prevention Measures on Adolescent Growth in Italy
Source: Healthcare (Basel). 2023 Jul 24;11(14):2101. doi: 10.3390/healthcare11142101 (PMC10379597; doi:10.3390/healthcare11142101)
Supplement: Supplementary file 1 [file healthcare-11-02101-s001.zip › healthcare-2500964-supplementary.pdf]

SUPPLEMENTARY MATERIAL

Assessing the impact of COVID-19 prevention measures on adolescent growth in Italy

Luciana Zaccagni, Natascia Rinaldo, Gianni Mazzoni, Simona Mandini, Sabrina Masotti, Stefania Toselli, Federica De Luca, and Emanuela Gualdi-Russo

Table S1. Basic socio-demographic information collected during the survey.

	**List of questions**
1.	Survey date
2.	School section
3.	Name
4.	Surname
5.	Gender F□ ; M□
6.	Date of birth (month/day/year)
7.	Place of birth

Table S2. Instructions to assess the Body image perception using the scale of silhouettes developed by Childress et al. [33].

	**Instructions**
1.	Among the eight silhouettes on the scale, circle the one that most closely resembles you.
2.	Among the same eight silhouettes, underline the number of the one with the look you would like to have.
